# Whole Genome Sequence of Polyresistant *Mycobacterium tuberculosis* CWCFVRF PRTB 19 Sputum Isolate from Chennai, India, Closely Clustering with East African Indian 5 Genogroup

**DOI:** 10.1128/genomeA.00702-14

**Published:** 2014-07-17

**Authors:** Dhanurekha Lakshmipathy, Umashankar Vetrivel, Gayathri Ramasubban, Lily Therese Kulandai, Hajib Narahari Madhavan, R. Sridhar, N. Meenakshi

**Affiliations:** a L&T Microbiology Research Centre, Vision Research Foundation, Chennai, India; b Centre for Bioinformatics, Vision Research Foundation, Chennai, India; c Department of Thoracic Medicine, Stanley Medical College, Chennai, India; d Institute of Thoracic Medicine, Chetpet, Chennai, India

## Abstract

We announce the draft genome sequence of a polyresistant *Mycobacterium tuberculosis* strain (CWCFVRF PRTB 19) isolated from the sputum of a clinically suspected tuberculosis patient, and it closely clusters to the East African Indian 5 (EAI5) lineage.

## GENOME ANNOUNCEMENT

Drug resistance in *Mycobacterium tuberculosis* is mainly due to the mutations in relatively restricted regions of the genome (1, 2). *M. tuberculosis* resistance to antituberculosis drugs has emerged as a serious problem. The management of tuberculosis relies on strong laboratory investigations and trained and dedicated personnel for treatment oversight and supervision. Treatment should be individualized for each patient on the basis of *in vitro* susceptibility pattern to antituberculosis drugs. In general, *M. tuberculosis* that exhibits *in vitro* drug resistance to more than one antituberculosis drug (either isoniazid or rifampin) is referred to as a polyresistant strain. Though the detection of mutations in the *M. tuberculosis* target genes by PCR-based DNA technology plays an important role in the detection of drug resistance, it will not provide a holistic view of the genomic evolution leading to drug resistance. Hence, there is a need for whole-genome sequencing technology to explore the complete mechanism of drug resistance emerging in *M. tuberculosis* strains.

We announce here the draft genome sequence of a polyresistant sputum isolate of *M. tuberculosis*, strain CWCFVRF PRTB 19, isolated from a patient clinically suspected to have tuberculosis. The isolate was found to be phenotypically resistant to streptomycin, isoniazid, ethambutol, and pyrazinamide by using the Bactec microMGIT culture system. Whole-genome sequencing was performed using an Ion Torrent PGM platform, similarly to in our previous work (3).

The generated sequence reads with a Phred score cutoff of ≥20 were *de novo* assembled using MIRA Assembler 3.4.1.1, which resulted in 115 contigs of 4,332,844 bp sequence length, with 130.93× coverage and an *N*_50_ of 81,962 bp. These sequences were further reordered to *M. tuberculosis* H37Rv (accession no. NC_000962.3) using Mauve (4) and in-house written scripts. The assembled sequences were annotated by NCBI PGAAP (http://www.ncbi.nlm.nih.gov/genomes/static/Pipeline.html), which revealed 4,058 protein-coding genes and 54 RNA-coding genes. The genome was also computationally spoligotyped using the SpolPred program (5), which resulted in the spoligotype octal code 075067737003511. Further spotclust analysis revealed that the strain belongs to a novel spoligotype closely clustering to East African Indian lineage 5 (EAI5), with a spotclust (6) probability of 0.99. This genome analysis will pave the way to understanding the epidemiology and genetic variations (mutations/polymorphisms) occurring in the polyresistant *M. tuberculosis* strains circulating in Chennai, India.

### Nucleotide sequence accession numbers.

This whole-genome shotgun project has been deposited at DDBJ/EMBL/GenBank under the accession no. JMIM00000000. The version described in this paper is version JMIM00000000.1.

## References

[1] Ramaswamy S Musser JM 1998 Molecular genetic basis of antimicrobial agent resistance in *Mycobacterium tuberculosis*: 1998 update. Tuber. Lung Dis. 79:3–29. 10.1054/tuld.1998.0002 10645439

[2] Zhang Y Telenti A 2000 Genetics of drug resistance in *Mycobacterium tuberculosis*, p 235–254 *In* Hatfull GF Jacobs WR Jr (ed), Molecular genetics of mycobacteria. ASM Press, Washington, DC

[3] Lakshmipathy D Vetrivel U Irudayam LT Ramasubban G Madhavan HN Sridhar R Meenakshi N 2014 Draft genome sequence of multidrug-resistant *Mycobacterium tuberculosis* strain CWCFVRF MDRTB 670, isolated from the sputum of a patient from Chennai, India, with clinically suspected tuberculosis. Genome Announc. 2(3):e00475-14. 10.1128/genomeA.00475-14 24855307 PMC4031345

[4] Darling AC Mau B Blattner FR Perna NT 2004 Mauve: multiple alignment of conserved genomic sequence with rearrangements. Genome Res. 14:1394–1403. 10.1101/gr.2289704 15231754 PMC442156

[5] Coll F Mallard K Preston MD Bentley S Parkhill J McNerney R Martin N Clark TG 2012 SpolPred: rapid and accurate prediction of Mycobacterium tuberculosis spoligotypes from short genomic sequences. Bioinformatics 28:2991–2993. 10.1093/bioinformatics/bts544 23014632 PMC3496340

[6] Vitol I Driscoll J Kreiswirth B Kurepina N Bennett KP 2006 Identifying *Mycobacterium tuberculosis* complex strain families using spoligotypes. Infect. Genet. Evol. 6:491–504. 10.1016/j.meegid.2006.03.003 16632413

